# Supplementary material for: Multi-Omics Analysis Reveals the Regulatory Mechanism of Probiotics on the Growth Performance of Fattening Sheep
Source: Animals (Basel). 2024 Apr 24;14(9):1285. doi: 10.3390/ani14091285 (PMC11083020; doi:10.3390/ani14091285)
Supplement: Supplementary file 1 [file animals-14-01285-s001.zip › Supplementary Materials.pdf]

Supplementary Materials

Table S1. Effects of different probiotics on growth performance of fattening sheep

**Item**	**CON**	**Bacillus licheniformis group**	**Bacillus subtilis binary group**	**Bifidobacterium triple group**	**Bifidobacterium tetravalent group**
ADG[1], kg/d	0.21±0.01	0.23±0.01	0.23±0.02	0.24±0.01	0.27±0.02
ADFI[2], kg/d	1.20±0.07	1.22±0.10	1.21±0.08	1.22±0.12	1.20±0.07
FCR[3]	0.18±0.01	0.19±0.01	0.19±0.01	0.20±0.01	0.23±0.01

[1]Average daily gain

[2]Feed conversion rate

[3]Average daily feed intake

Table S2. Significant differences in metabolites of rumen fluid between PRB and CON groups in fattening sheep

Name	MZ[1]	R．T[2]（min）	VIP[3]	Pvalue[4]	FC[5]
**Negative**					
Phenol	93.036	38.397	1.475	0.024	2.231
p-Cresol	107.050	45.147	1.668	0.006	1.548
Pyrocatechol	109.029	46.098	1.641	0.006	2.696
L-Proline	114.054	211.696	1.747	0.035	0.258
Indole	116.051	206.308	1.287	0.027	2.414
2-Hydroxy-3-methylbutyric acid	117.056	223.817	1.651	0.007	1.487
L-Norleucine	130.087	150.238	1.793	0.030	0.236
5-Hydroxyhexanoic acid	131.071	190.973	1.384	0.008	1.576
Anthranilic acid (Vitamin L1)	136.040	340.703	1.480	0.010	1.556
Salicylic acid	137.025	38.421	1.478	0.023	2.258
2-Hydroxyphenylacetic acid	151.040	184.461	1.564	0.007	0.561
3-Hydroxyphenylacetic acid	151.040	45.070	1.453	0.018	1.375
p-Hydroxyphenylacetic acid	151.040	171.694	1.356	0.013	1.385
L-Cysteinesulfinic acid	152.002	68.174	1.757	0.012	1.858
Gentisic acid	153.019	68.628	1.614	0.004	1.608
3,3-Dimethylglutaric acid	159.066	82.255	1.954	0.000	1.737
L-Methionine S-oxide	164.038	353.148	1.516	0.024	0.547
Uric acid	167.020	302.720	1.725	0.029	0.528
Suberic acid	173.081	329.530	1.262	0.048	1.250
N2-Acetyl-L-ornithine	173.092	345.316	1.540	0.025	0.560
4-Pyridoxic acid	182.046	40.633	1.349	0.021	1.368
Azelaic acid	187.098	315.988	1.631	0.008	1.340
5-Hydroxyindoleacetate	190.050	199.693	1.765	0.010	2.507
Perseitol	193.069	157.362	1.793	0.039	0.116
Sebacic acid	201.113	300.962	1.571	0.009	1.308
Pantothenol	204.123	75.948	1.657	0.002	1.604
p-Hydroxycinnamaldehyde	207.066	43.848	1.656	0.007	1.431
resorcinol	219.065	33.583	1.763	0.001	0.601
Xanthurenic acid	221.056	120.423	1.492	0.009	1.290
N-Acetyl-L-tyrosine	222.076	204.511	1.521	0.017	0.604
Tropic acid	225.075	330.730	1.988	0.001	2.394
Deoxycytidine	227.091	187.353	1.853	0.002	2.096
Traumatic Acid	227.128	181.055	1.551	0.028	1.505
d-Dethiobiotin	230.148	270.142	1.480	0.015	1.374
L-Cystine	239.015	165.249	1.799	0.001	0.620
Lumichrome	241.070	66.168	1.546	0.002	1.410
Pentobarbital	242.146	160.486	1.380	0.025	1.545

Nicotinamide	243.086	103.757	1.968	0.001	2.384
Zingerone	253.106	45.158	1.372	0.049	1.816
D-Glucosamine 1-phosphate (Glucosamine-1P)	258.037	391.772	1.217	0.022	1.282
Coumestrol	267.028	43.948	1.646	0.003	0.714
Phloretin	273.075	46.518	2.538	0.049	1.250
D-Neopterin	274.062	76.572	1.363	0.043	1.634
Niflumic Acid	281.055	106.464	1.812	0.001	1.332
12-Oxo-2,3-dinor-10,15-phytodienoic acid	285.149	178.096	1.957	0.000	1.656
Nname,cis-9,10-Epoxystearic acid	297.241	66.948	1.261	0.035	0.607
N1-Methyl-2-pyridone-5-carboxamide	303.105	27.580	1.788	0.002	1.802
2E-Eicosenoic acid	309.279	41.938	1.276	0.047	0.596
Prunasin	316.086	302.320	1.190	0.040	1.508
Hesperetin	323.053	113.784	1.480	0.035	1.744
2-Amino-3-methoxy-benzoic acid	333.107	152.105	1.879	0.000	1.668
Flutamide	335.088	38.454	1.843	0.000	0.656
Erucic acid	337.309	41.691	1.264	0.047	0.586
Sphinganine	338.255	25.674	1.581	0.045	0.451
Stearic acid	343.282	48.450	1.669	0.006	0.709
Adenosine 3'-monophosphate	346.053	390.849	1.431	0.037	0.644
5(S)-HpETE	395.253	154.766	1.947	0.000	1.752
1-Palmitoyl-2-oleoyl-phosphatidylglycerol	747.514	47.200	1.767	0.024	0.142
2-Oleoyl-1-palmitoyl-sn-glycero-3-phosphocholine(PC(16:0/18:1(9Z)))	758.562	132.080	1.904	0.003	0.459
Ginsenoside Rg3	783.487	144.285	1.583	0.002	0.637
**Postive**					
Diethanolamine	70.067	299.565	1.429	0.006	0.565
Glutaraldehyde	83.050	371.407	1.163	0.031	0.772
L-Alanine	90.056	333.993	1.128	0.039	0.737
3,3-Dimethylacrylic acid	101.060	371.407	1.221	0.026	0.716
L-Proline	116.072	299.692	1.496	0.003	0.548
5-Aminopentanoic acid	118.086	386.709	1.071	0.032	0.749
3-Aminobenzoic acid	120.045	43.681	1.495	0.002	1.293
Nicotinamide	123.056	63.454	1.092	0.028	0.590
Quinone	126.055	286.965	1.576	0.007	0.565
1,4-Dihydroxybenzene	128.071	79.520	1.699	0.000	1.515
L-Pipecolic acid	130.087	264.703	1.414	0.020	3.522
3-Ureidopropionate	133.061	295.565	1.265	0.035	0.471
Cyclohexylammonium	138.066	50.561	1.129	0.008	0.440
DL-2-Aminoadipic acid	144.065	280.845	1.743	0.004	0.336
N,N-Bis(2-hydroxyethyl)glycine	146.081	110.586	1.640	0.001	1.507

(3-Carboxypropyl)trimethylammonium cation	146.119	364.221	1.367	0.016	1.650
Dimethylbenzimidazole	147.092	46.307	1.749	0.000	0.378
D-Lyxose	150.055	104.018	1.547	0.001	1.922
Phenylephrine	150.091	236.31	1.183	0.049	0.679
N-Acetylglutamine	153.066	309.700	1.116	0.046	1.353
L-Valine	159.113	246.734	1.499	0.005	0.366
trans-2-Hydroxycinnamic acid	165.054	288.816	1.171	0.046	0.715
DL-Methionine sulfoxide	166.051	366.342	1.444	0.031	0.478
1-Methylxanthine	167.053	41.177	1.582	0.009	1.595
Pyridoxal (Vitamin B6)	168.065	104.494	1.361	0.029	1.653
Pyridoxine	170.081	120.135	1.542	0.019	3.428
L-Norleucine	173.126	258.615	1.728	0.000	0.541
Ethyl hydrogen malonate	174.076	101.712	1.284	0.042	1.482
N-Carboxyethyl-.gamma.-aminobutyric acid	176.092	366.400	1.577	0.012	2.658
(Z)-4-Decen-1-ol	179.143	178.522	1.747	0.000	1.859
Dacarbazine	183.099	111.260	1.509	0.004	1.411
4-Pyridoxic acid	184.061	41.190	1.673	0.006	2.033
Ala-Leu	185.128	241.216	1.743	0.014	16.798
Homogentisic acid	186.076	287.134	1.548	0.009	0.613
Hydroxyacetone	187.036	277.060	1.427	0.029	2.735
2-Methylglutaric acid	188.092	323.008	1.667	0.000	1.568
4-Hydroxybutanoic acid lactone	190.107	358.502	1.381	0.017	1.351
Ornithine	196.105	132.256	1.443	0.019	2.503
N-Acetyl-L-Histidine	198.087	293.339	1.530	0.005	0.523
.beta.-Citronellol	201.123	321.480	1.377	0.032	2.162
3-Hydroxybenzoate	202.044	231.489	1.334	0.013	0.973
3-Methoxy-4-hydroxyphenylethyleneglycol	202.108	344.396	1.492	0.013	1.393
Xanthurenic acid	206.043	41.929	1.585	0.003	1.633
Pantothenol	206.138	76.634	1.663	0.008	1.861
4-Aminobutyric acid	207.137	47.113	1.363	0.028	1.695
Dihydrolipoate (dihydrolipoic acid)	208.063	280.953	1.742	0.005	0.313
3-Methoxytyramine	209.128	403.358	1.288	0.025	0.642
2,2-Dimethyl Succinic acid	210.076	127.554	1.695	0.000	1.662
Jasmonic acid	211.133	181.583	1.335	0.010	1.291
Limonene-1,2-epoxide	213.148	50.191	1.250	0.026	0.453
Pro-Thr	216.105	369.882	1.408	0.035	1.555
N-.alpha.-Acetyl-L-arginine	217.124	320.733	1.488	0.007	1.516
6-Benzylaminopurine	226.107	51.093	1.150	0.038	1.514
Desoxypeganine	233.128	194.622	1.416	0.003	1.779
Indoleacetic acid	236.091	345.116	1.383	0.018	1.575

N-Formylmethionine	238.070	180.435	1.079	0.044	0.797
Gly-Val	238.116	296.570	1.792	0.000	1.990
.gamma.-L-Glu-.epsilon.-L-Lys	240.137	34.912	1.404	0.025	0.435
Nicotinuric acid	241.082	320.768	1.217	0.034	1.212
Thymidine	243.097	102.313	1.220	0.046	2.330
Gly-Gln	248.064	173.779	1.226	0.021	2.267
Myristic acid	251.200	107.326	1.467	0.004	0.481
4-Hydroxybenzaldehyde	262.110	196.019	1.217	0.019	2.048
Coumestrol	269.043	45.321	1.747	0.000	0.432
Formononetin	269.080	40.412	1.163	0.031	0.654
Larixinic Acid	270.097	388.756	1.628	0.001	1.551
Met-Gln	277.110	286.803	1.496	0.002	0.586
alpha-Linolenic acid	279.231	41.675	1.587	0.000	0.247
1-Methyladenosine	282.119	284.845	1.173	0.037	0.777
Temazepam	283.059	42.872	1.531	0.001	0.472
trans-Vaccenic acid	283.262	38.648	1.505	0.001	0.245
Glycerol 1-myristate	285.241	46.109	1.622	0.000	0.327
Val-Met	290.146	296.286	1.604	0.000	1.742
Procaterol	291.169	239.327	1.613	0.001	1.556
Ergothioneine	294.117	286.832	1.547	0.002	0.588
Lys-Ser	300.095	272.294	1.125	0.042	0.750
Retinol (Vitamin A)	304.262	45.472	1.812	0.005	0.182
L-Anserine	307.070	45.231	1.124	0.002	0.503
Phenylacetic acid	311.065	37.475	1.571	0.001	0.584
16-hydroxy hexadecanoic acid	314.276	46.195	1.639	0.000	0.298
Gln-Asn	321.140	240.220	1.248	0.033	0.767
(+-)8,9-DHET	321.241	47.027	1.416	0.005	0.695
Erucic acid	321.313	34.197	1.250	0.038	0.452
Met-Glu	323.058	126.665	1.556	0.001	2.102
3-Methylphenylacetic acid	323.123	149.091	1.548	0.001	1.414
Diethyl sebacate	325.142	38.576	1.556	0.001	0.548
Phe-Pro	326.147	39.037	1.691	0.000	0.455
Lys-Trp	332.190	365.621	1.762	0.000	1.894
Benzylbutylphthalate	335.125	43.298	1.438	0.003	0.548
N-Tigloylglycine	337.142	36.692	1.622	0.000	0.421
Tyr-Asp	338.131	313.025	1.750	0.000	2.087
20-Hydroxyarachidonic acid	338.269	44.462	1.296	0.007	0.555
Erucamide	338.342	34.253	1.178	0.045	0.446
Arg-Gln	341.137	42.710	1.347	0.004	0.581
Adenosine 3'-monophosphate	348.069	391.611	1.283	0.029	0.614
L-Fucose	351.121	37.354	1.699	0.000	0.336
Arg-Tyr	360.169	38.334	1.778	0.000	0.534
Bisdemethoxycurcumin	369.131	39.145	1.287	0.007	0.575
Lithocholic acid	399.287	35.143	1.144	0.029	0.567

.delta.-Tocopherol	402.344	33.355	1.677	0.000	0.370
Desmosterol	407.329	36.622	1.744	0.000	0.465
Lathosterol	409.344	34.995	1.816	0.000	0.457
Lanosterol	409.380	32.825	1.261	0.044	0.343
gamma-Tocopherol	416.361	33.061	1.673	0.000	0.369
Dihydrotachysterol	421.344	34.151	1.805	0.000	0.251
6"-O-Acetyldaidzin	423.116	50.422	1.645	0.001	0.640
N-Acetyl-D-lactosamine	428.122	46.245	1.353	0.009	0.703
Hexacosanoic acid	441.370	34.368	1.772	0.000	0.388
Uvaol	443.384	32.823	1.654	0.000	0.360
Enoxolone	453.333	48.639	1.669	0.000	0.532
1-Palmitoyl-2-hydroxy-sn-glycero-3-phosphoethanolamine	454.292	187.791	1.329	0.011	0.341
Ascorbic acid 6-palmitate	456.296	187.872	1.421	0.008	0.395
Ergocalciferol (Vitamin D2)	457.364	34.589	1.768	0.000	0.311
25-Hydroxycholesterol	463.376	33.061	1.783	0.000	0.228
Bufexamac	464.276	45.480	1.523	0.011	0.265
1-Myristoyl-sn-glycero-3-phosphocholine	468.306	196.203	1.243	0.032	0.366
Cyclopamine	472.347	47.816	1.5009	0.022	0.442
1-Stearoyl-2-hydroxy-sn-glycero-3-phosphoethanolamine	482.321	184.309	1.252	0.025	0.385
Verapamil	490.288	45.375	1.275	0.045	0.468
1-Palmitoyl-sn-glycero-3-phosphocholine	496.338	181.629	1.435	0.034	0.239
Pristimerin	528.303	159.169	1.408	0.033	0.237
Bata-Carotene	536.427	130.761	1.708	0.002	0.320
1-O-(cis-9-Octadecenyl)-2-O-acetyl-sn-glycero-3-phosphocholine	551.388	141.262	1.175	0.024	0.486
Geranylgeraniol	619.486	45.372	1.370	0.009	0.376
1-Palmitoyl-2-oleoyl-sn-glycero-3-phosphoethanolamine	718.533	46.126	1.717	0.000	0.299
PC(16:0/16:0)	756.549	131.846	1.872	0.001	0.062
Thioetheramide-PC	774.559	141.189	1.837	0.003	0.058
Sphingomyelin (d18:1/18:0)	775.555	45.352	1.812	0.000	0.216

[1]MZ = mass-to-charge ratio.

[2]R.T = represents retention time.

[3]VIP >1 and [4]P-value<0.05 are listed in the table. P -values were calculated according to Student's T-test (n=6).

[5]FC = fold change. If the fold change value is less than 1, it means that there is less metabolite in the PRB group than in the CON group.

Table S3. Pathway analysis of rumen fluid metabolomics in PRB and CON groups of fattening sheep

Pathway	Total	Hits[1]	Raw P[2]	Impact[3]	Hits compounds
Vitamin B6 metabolism	9	3	0.003	0.569	Pyridoxine cpd:C00314; Pyridoxal cpd:C00250; 4-Pyridoxic acid cpd:C00847
Tyrosine metabolism	42	4	0.050	0.049	Metanephrine cpd:C05588; 3-Methoxytyramine cpd:C05587; Homogentisic acid cpd:C00544; Vanylglycol cpd:C05594
Riboflavin metabolism	11	2	0.051	0.333	Hydroquinone cpd:C00530; Quinone cpd:C00472
Pantothenate and CoA biosynthesis	15	2	0.089	0.041	Ureidopropionic acid cpd:C02642; L-Valine cpd:C00183
Ubiquinone and other terpenoid-quinone biosynthesis	3	1	0.098	0	Homogentisic acid cpd:C00544
beta-Alanine metabolism	17	2	0.110	0.222	Ureidopropionic acid cpd:C02642; Anserine cpd:C01262
Steroid biosynthesis	35	3	0.111	0.205	Lathosterol cpd:C01189; Lanosterin cpd:C01724; Desmosterol cpd:C01802
alpha-Linolenic acid metabolism	9	1	0.267	1	Alpha-Linolenic acid cpd:C06427
Valine, leucine and isoleucine biosynthesis	11	1	0.316	0.333	L-Valine cpd:C00183
Pyrimidine metabolism	37	2	0.358	0.021	Ureidopropionic acid cpd:C02642; Thymidine cpd:C00214
Nicotinate and nicotinamide metabolism	13	1	0.362	0.159	Niacinamide cpd:C00153
Biosynthesis of unsaturated fatty acids	42	2	0.419	0	Erucic acid cpd:C08316; Alpha-Linolenic acid cpd:C06427
Fructose and mannose metabolism	19	1	0.482	0	L-Fucose cpd:C01019
Lysine degradation	20	2	0.500	0	Aminoadipic acid cpd:C00956

Aminoacyl-tRNA biosynthesis	64	1	0.648	0	L-Valine cpd:C00183; L-Proline cpd:C00148
Amino sugar and nucleotide sugar metabolism	37	1	0.724	0	L-Fucose cpd:C01019
Fatty acid biosynthesis	38	2	0.734	0	Myristic acid cpd:C06424
Valine, leucine and isoleucine degradation	38	1	0.734	0	L-Valine cpd:C00183
Tryptophan metabolism	41	1	0.761	0	Indoleacetic acid cpd:C00954
Arginine and proline metabolism	44	1	0.785	0.078	L-Proline cpd:C00148
Primary bile acid biosynthesis	46	1	0.800	0.011	25-Hydroxycholesterol cpd:C15519
Cysteine and methionine metabolism	28	2	0.141	0.023	L-Cystine cpd:C00491; 3-Sulfinoalanine cpd:C00606
Taurine and hypotaurine metabolism	7	1	0.155	0.250	3-Sulfinoalanine cpd:C00606
Phenylalanine metabolism	9	1	0.195	0	Ortho-Hydroxyphenylacetic acid cpd:C05852
Sphingolipid metabolism	21	1	0.398	0.143	Sphinganine cpd:C00836
Purine metabolism	68	1	0.812	0.008	Uric acid cpd:C00366

[1] Hits represent the number of significantly different ruminal metabolites matched in one pathway.

[2] P is the original P value obtained by pathway analysis.

[3] Impact is the influencing factor of the pathway obtained by topology analysis.

Table S4. Significant differences in metabolites between PRB group and CON group in serum of fattening sheep

Name	MZ	R.T(min)	VIP	Pvalue	FC
**Negative**					
Pyruvate	87.001	121.770	1.789	0.029	2.186
4-Nitrophenol	138.019	38.009	2.151	0.000	0.563
2-Hydroxyadenine	167.071	48.673	1.460	0.046	2.080
Glycerol 3-phosphate	171.006	372.989	2.136	0.001	0.588
Urocanic acid	174.995	24.518	1.921	0.018	3.985
L-Ascorbic acid	175.024	294.968	1.992	0.012	2.044
Allantoate/Allantoic acid	175.047	333.298	2.337	0.000	0.282
Beta-Alanine	177.091	48.385	1.997	0.002	0.411
Hydroxyphenyllactic acid	181.050	177.757	1.488	0.030	1.460
Dihydrothymine	187.072	372.274	2.169	0.000	0.296
D-galacturonic acid	193.035	362.862	1.406	0.047	0.914
Citramalic acid	207.050	287.748	1.490	0.036	1.977
L-Kynurenine	207.077	260.097	1.690	0.034	0.712
Indoxyl sulfate	212.001	33.384	1.881	0.001	0.552
Pyrocatechol	219.065	34.008	1.798	0.038	1.655
Lipoamide	221.081	47.500	1.959	0.018	0.185
Pindone	229.088	186.235	1.965	0.040	2.546
Suberylglycine	230.102	326.586	1.508	0.021	0.612
Glucosamine	238.092	296.552	1.809	0.007	0.658
Phosphorylcholine	242.079	373.433	2.311	0.000	0.596
Stavudine	245.056	167.719	2.016	0.001	0.384
Muramic acid	250.093	352.874	1.532	0.019	0.495
5'-O-methylthymidine	256.104	144.418	2.136	0.008	0.128
(R)-mevalonic acid 5-Phosphate	265.089	26.224	1.824	0.033	3.814
N4-Acetylcytidine	284.088	158.482	2.206	0.000	4.718
N-Acetylneuraminic acid	290.087	325.584	1.934	0.002	0.873
Mevalonic acid	295.139	316.777	1.128	0.039	0.679
3'-O-methylguanosine	296.099	189.024	1.546	0.034	1.213
Eicosapentaenoic Acid	301.216	43.507	1.482	0.027	0.794
Nicotinamide ribotide	333.057	408.698	1.397	0.028	0.803
Behenic acid	339.326	40.951	1.450	0.017	0.758
Sucrose	342.118	315.467	1.916	0.001	0.322
Maltitol	344.113	260.680	1.986	0.001	0.449
Tricosanoic acid	353.341	40.545	1.303	0.022	0.734
Tetracosanoic acid	367.356	40.381	1.666	0.002	0.646
Hexacosanoic acid	395.387	39.835	1.557	0.007	0.555
Fludrocortisone acetate	403.195	259.111	1.638	0.032	3.062
1-Palmitoyl Lysophosphatidic Acid	409.234	180.801	1.371	0.036	0.849

alpha-Tocopherol (Vitamin E)	429.371	31.841	1.462	0.036	2.004
N-Formylmethionyl-Leucylphenylalanine	436.197	294.122	1.575	0.001	0.334
1-Stearoyl-sn-glycerol 3-phosphocholine	522.353	177.094	1.418	0.025	0.745
MK 571	573.126	333.185	1.856	0.002	0.237
**Postive**					
Glycine	76.039	368.020	1.935	0.000	0.420
Diethanolamine	88.076	211.625	2.175	0.000	21.985
L-Alanine	90.055	334.322	1.423	0.024	0.906
Glutaraldehyde	101.059	371.458	1.704	0.001	0.876
Betaine aldehyde	102.090	343.589	1.301	0.036	0.816
N-Methylhydantoin	115.049	334.322	1.579	0.003	0.889
Acetylglycine	118.049	368.037	2.030	0.000	0.313
Creatine	132.077	334.322	1.369	0.038	0.898
Nicotinamide N-oxide	139.049	150.762	1.297	0.023	0.679
Larixinic Acid	144.064	252.795	1.927	0.000	0.288
Pyruvaldehyde	145.049	293.416	1.778	0.040	2.011
Levonordefrin	148.074	171.378	1.432	0.014	0.692
DL-a-Hydroxybutyric acid	149.022	33.168	1.914	0.000	0.480
2-Ethoxyethanol	151.095	63.928	1.567	0.006	0.598
Acetyl-DL-Leucine	156.100	390.035	1.605	0.011	0.686
Caproic acid	158.117	47.430	1.707	0.010	2.156
Acetyl-DL-Valine	160.096	313.750	1.238	0.042	0.516
Cyclohexylamine	160.133	371.359	1.319	0.016	0.809
trans-2-Octenoic acid	160.133	387.590	1.417	0.007	0.806
1-Aminocyclopropanecarboxylic acid	162.075	252.795	1.776	0.001	0.483
L-Carnitine	162.111	292.158	1.139	0.047	0.844
beta-Hydroxybutyrate	168.065	252.499	1.920	0.000	0.337
L-Pipecolic acid	171.112	252.565	1.987	0.006	0.191
.beta.-Cyano-L-alanine	175.070	209.013	1.596	0.013	0.642
L-Citrulline	176.102	393.110	1.536	0.005	0.715
3,4-Dihydroxyphenylacetic acid	186.075	252.614	1.792	0.000	0.319
Valproic acid	186.174	37.645	1.954	0.000	1.872
Kynuramine	187.089	283.929	1.951	0.000	0.195
L-Arabinose	192.087	47.551	1.059	0.043	0.625
Oxindole	194.080	204.577	1.475	0.029	0.749
Ala-Ala	202.117	267.679	1.331	0.043	0.686
Pyridostigmine cation	204.086	252.598	1.998	0.000	0.362
Acetylcarnitine	204.123	293.416	1.826	0.043	2.533
Pelletierine	205.126	293.423	1.840	0.046	2.617
Pyridoxine	211.106	85.616	1.602	0.002	0.552
DL-Vanillylmandelic acid	216.085	352.428	1.530	0.020	0.586
N-.alpha.-Acetyl-L-arginine	217.128	355.666	1.677	0.019	0.612

Thr-Val	218.123	390.979	1.840	0.001	0.513
D-Glucono-1,5-lactone	220.080	394.621	1.489	0.004	0.470
His-Ser	225.097	303.662	1.732	0.025	2.342
D-gluconate	238.091	368.003	2.022	0.000	0.257
Pro-Phe	245.123	292.865	1.512	0.045	0.521
Gly-Glu	246.107	365.740	1.941	0.000	0.495
Val-Gln	246.144	313.231	2.013	0.000	0.134
Glycerophosphocholine	258.110	372.063	1.954	0.001	0.557
2-Methyl-3-hydroxybutyric acid	259.119	28.241	1.368	0.025	1.489
1,7-Dimethyluric acid	260.073	370.201	2.017	0.000	0.325
Flumequine	261.076	370.429	1.982	0.000	0.536
Phthalic acid Mono-2-ethylhexyl Ester	279.158	33.252	2.106	0.000	0.418
Val-Tyr	280.138	313.221	1.753	0.000	0.377
Phenylethylamine	281.136	37.061	1.261	0.042	1.680
Ile-Ser	282.142	313.196	1.892	0.000	0.558
Ile-Asn	284.105	371.607	2.026	0.004	0.642
N4-Acetylcytidine	286.102	158.142	1.945	0.001	3.467
Ile-Arg	288.202	323.000	1.363	0.032	0.779
Lys-Cys	291.154	464.655	1.290	0.044	0.669
Gamma-Glutamylcysteine	292.101	409.542	1.785	0.002	0.591
gamma-L-Glutamyl-L-phenylalanine	295.127	323.362	1.197	0.024	0.789
Gly-Arg	295.149	433.332	1.579	0.005	0.441
16-Hydroxypalmitic acid	295.225	47.610	1.959	0.006	1.567
Ile-Thr	296.158	294.156	1.713	0.002	0.526
4-Oxoretinol	301.215	35.353	1.742	0.004	1.661
Lys-Asn	305.119	326.400	1.218	0.038	0.618
Tyr-Lys	309.164	439.953	1.768	0.002	0.437
Omeprazole	310.094	364.899	1.915	0.001	0.400
Pro-Met	310.112	409.319	1.884	0.000	0.373
Phe-Cys	310.127	280.996	1.817	0.000	0.464
Tyr-Phe	311.132	370.987	2.099	0.000	0.397
Met-Tyr	312.113	306.241	1.735	0.001	0.374
Argininosuccinic acid	313.113	306.241	1.782	0.000	0.422
His-Tyr	318.128	410.891	1.856	0.000	0.501
Zolmitriptan	326.125	434.955	1.958	0.000	0.469
N-Oleoylethanolamine	326.304	35.303	1.509	0.038	1.413
D-Ribulose 1,5-bisphosphate	328.017	352.737	1.469	0.028	0.615
Phe-Tyr	328.138	280.875	1.823	0.000	0.358
(-)-Medicarpin	331.110	372.176	2.008	0.000	0.332
beta-Octylglucoside	337.159	354.300	1.954	0.000	0.336
Arg-Tyr	337.172	450.647	1.947	0.000	0.382
Arg-Cys	338.154	393.075	1.969	0.000	0.333
Enoxacin	338.162	353.817	2.001	0.000	0.383
Arg-Thr	339.175	451.272	1.993	0.000	0.452

Met-Met	344.099	323.627	1.730	0.001	0.463
Famciclovir	344.133	315.326	1.851	0.001	0.298
Lomefloxacin	352.146	295.811	1.817	0.003	1.523
Visnadin	353.132	293.863	2.004	0.000	0.315
(+-)5,6-DHET	356.277	83.476	1.947	0.008	0.155
Behenic acid	358.366	51.570	1.231	0.014	0.507
Cortisone	361.199	43.327	1.478	0.043	1.596
20-Hydroxyarachidonic acid	362.268	43.412	1.508	0.028	0.606
Phenoxybenzamine	367.148	282.726	1.874	0.000	0.318
Tyr-Glu	371.143	294.147	2.046	0.003	0.254
Cortexolone	385.170	364.673	1.732	0.007	0.452
Dioctyl phthalate	391.283	32.456	1.291	0.049	1.386
Troglitazone	459.206	338.329	2.073	0.012	0.082
1-Stearoyl-2-arachidonoyl-sn-glycerol	627.532	46.640	2.196	0.000	0.126
PC(20:5(5Z,8Z,11Z,14Z,17Z)/20:5(5Z,8Z,11Z,14Z,17Z))	809.542	46.498	1.737	0.044	66.192

Table S5. Pathway analysis of serum metabolomics in PRB and CON groups of fattening sheep

Pathway	Total	Hits	Raw P	Impact	Hits compounds
Glycine, serine and threonine metabolism	32	4	0.002	0.292	Glycine cpd:C00037; Betaine aldehyde cpd:C00576; Creatine cpd:C00300; Pyruvaldehyde cpd:C00546
Arginine and proline metabolism	44	3	0.046	0.061	Citrulline cpd:C00327; Argininosuccinic acid cpd:C03406; Creatine cpd:C00300
Cyanoamino acid metabolism	6	1	0.107	0	Glycine cpd:C00037
Methane metabolism	9	1	0.157	0	Glycine cpd:C00037
Nitrogen metabolism	9	1	0.157	0	Glycine cpd:C00037
Vitamin B6 metabolism	9	1	0.157	0.078	Pyridoxine cpd:C00314
Phenylalanine metabolism	9	1	0.157	0.222	Phenylethylamine cpd:C05332
Pentose and glucuronate interconversions	15	1	0.248	0	L-Arabinose cpd:C00259
Pentose phosphate pathway	19	1	0.303	0	Gluconolactone cpd:C00198
Propanoate metabolism	20	1	0.316	0	2-Hydroxybutyric acid cpd:C05984
Pyruvate metabolism	22	1	0.342	0.054	Pyruvaldehyde cpd:C00546
Alanine, aspartate and glutamate metabolism	23	1	0.354	0.020	Argininosuccinic acid cpd:C03406
Porphyrin and chlorophyll metabolism	25	1	0.379	0	Glycine cpd:C00037
Glutathione metabolism	26	1	0.390	0.006	Glycine cpd:C00037
Glycerophospholipid metabolism	29	1	0.425	0.024	Glycerophosphocholine cpd:C00670
Biosynthesis of unsaturated fatty acids	42	1	0.553	0	Behenic acid cpd:C08281
Tyrosine metabolism	42	1	0.553	0.001	3,4-Dihydroxybenzeneacetic acid cpd:C01161
Primary bile acid biosynthesis	46	1	0.586	0.030	Glycine cpd:C00037
Drug metabolism - cytochrome P450	56	1	0.660	0	Valproic acid cpd:C07185
Aminoacyl-tRNA biosynthesis	64	1	0.709	0	Glycine cpd:C00037

Steroid hormone biosynthesis	67	1	0.726	0.017	Cortisone cpd:C00762
Terpenoid backbone biosynthesis	15	2	0.020	0.317	Mevalonic acid cpd:C00418; Mevalonic acid-5P cpd:C01107
Pyrimidine metabolism	37	2	0.105	0.001	Dihydrothymine cpd:C00906; Beta-Alanine cpd:C00099
Ascorbate and aldarate metabolism	9	1	0.128	0	Ascorbic acid cpd:C00072
Valine, leucine and isoleucine biosynthesis	11	1	0.155	0	Pyruvic acid cpd:C00022
Nicotinate and nicotinamide metabolism	13	1	0.180	0	Nicotinamide ribotide cpd:C00455
Histidine metabolism	14	1	0.193	0.130	Urocanic acid cpd:C00785
Pantothenate and CoA biosynthesis	15	1	0.205	0	Beta-Alanine cpd:C00099
beta-Alanine metabolism	17	1	0.229	0.444	Beta-Alanine cpd:C00099
Glycerolipid metabolism	18	1	0.241	0.026	Pyruvic acid cpd:C00022
Butanoate metabolism	20	1	0.264	0	Pyruvic acid cpd:C00022
Citrate cycle (TCA cycle)	20	1	0.264	0.072	Pyruvic acid cpd:C00022
Glycolysis or Gluconeogenesis	26	1	0.329	0.099	Pyruvic acid cpd:C00022
Cysteine and methionine metabolism	28	1	0.350	0.021	Pyruvic acid cpd:C00022
Amino sugar and nucleotide sugar metabolism	37	1	0.435	0	Glucosamine cpd:C00329
Tryptophan metabolism	41	1	0.469	0.031	L-Kynurenine cpd:C00328
Purine metabolism	68	1	0.654	0.000	Allantoic acid cpd:C00499

Table S6. Significant differences in metabolites between PRB and CON groups in urine of fattening sheep

Name	MZ	R.T（min）	VIP	Pvalue	FC
**Negative**					
Propionic acid	73.030	135.036	1.383	0.015	0.254
Taurine	124.007	332.597	1.299	0.038	0.479
Ammeline	127.051	300.504	1.404	0.049	0.563
Dihydroxyfumarate	129.055	336.337	1.350	0.092	0.797
Xylitol	133.050	233.909	1.449	0.040	0.565
Hydroxyacetone	133.048	41.372	1.732	0.034	0.640
Ethosuximide	140.071	228.762	1.797	0.015	0.529
Cyanuric acid	145.036	96.040	1.337	0.046	2.153
Gentisic acid	153.019	145.061	1.518	0.025	0.464
3-Isopropylmalate	157.049	131.265	1.487	0.029	0.500
3-Dehydroshikimic acid	171.028	320.273	1.666	0.023	0.552
3-Phosphoserine	185.011	71.820	1.720	0.004	0.424
sn-Glycerol 1-phosphate	188.091	253.285	1.873	0.025	0.483
Salicyluric acid	194.046	160.015	1.561	0.031	0.400
Glucosaminic acid	194.067	265.377	1.441	0.041	0.550
N-Acetyl-L-aspartic acid	196.027	180.462	1.234	0.046	0.626
O-Phospho-L-threonine	199.028	51.153	1.511	0.024	0.458
L-Serine	290.080	192.562	1.638	0.021	0.538
5-L-Glutamyl-L-alanine	218.091	342.785	1.735	0.025	0.714
L-Carnosine	225.099	407.937	1.618	0.024	0.620
L-Gulonic gamma-lactone	237.056	28.630	1.608	0.031	0.604
4-Hydroxybenzaldehyde	243.069	46.267	1.446	0.011	0.547
gamma-Glutamyl-L-methionine	259.071	269.993	1.573	0.042	0.555
Isoproturon	265.152	134.308	1.840	0.022	0.309
Gentisaldehyde	275.057	33.303	1.490	0.028	0.561
3-Deoxy-2-keto-6-phosphogluconic acid	279.088	192.481	1.369	0.015	0.630
Uridine	281.017	296.587	1.804	0.026	0.545
5-methoxytryptophan	293.121	248.128	1.709	0.042	0.316
D-Ribulose 1,5-bisphosphate	309.986	28.091	1.726	0.032	0.650
Sulfaphenazole	313.072	67.821	1.376	0.042	0.535
beta-Octylglucoside	313.162	268.436	1.561	0.022	0.592
D-Mannitol 1-phosphate	321.167	105.877	1.376	0.038	0.550
Maltitol	325.111	393.745	1.482	0.024	0.664
Clozapine	325.128	46.372	1.749	0.002	0.481
Tosyllysine Chloromethyl Ketone	331.082	224.324	1.378	0.049	0.507

Phloretin	333.097	180.420	1.278	0.039	0.495
3'-O-methylguanosine	334.057	28.572	1.780	0.028	0.634
Dicumarol	335.052	102.248	1.452	0.016	0.601
Thiamine monophosphate	343.067	72.633	1.636	0.010	0.448
Phenolphthalein	355.045	45.751	1.565	0.036	0.674
Flavin mononucleotide (FMN)	455.095	47.191	1.559	0.022	0.571
UDP-D-Galactose	565..061	132.919	1.426	0.028	0.529
**Postive**					
Glutaraldehyde	83.048	372.746	1.819	0.004	0.596
Choline	104.106	148.043	1.748	0.013	0.661
4-Aminophenol	110.059	89.159	1.568	0.038	0.674
Cytosine	112.050	158.047	1.619	0.017	0.553
Benzamide	122.058	79.855	1.724	0.016	0.689
Taurine	126.022	281.584	1.320	0.027	0.525
5-Methylcytosine	126.065	188.997	1.947	0.025	0.371
4-Hydroxybutanoic acid lactone	128.070	78.507	1.377	0.043	0.728
Ethosuximide	142.085	229.273	1.771	0.008	0.661
Oxyquinoline	146.059	188.149	1.454	0.012	0.654
Diacetyl	150.054	44.796	1.517	0.031	0.560
Phenol	155.069	106.249	1.502	0.019	0.475
Caffeic Acid	163.041	169.290	1.454	0.047	0.690
Formylanthranilic acid	165.039	197.187	1.599	0.047	0.712
L-Canavanine	176.089	262.291	1.379	0.035	0.704
Succinate	179.051	33.044	1.358	0.045	0.612
4-Imidazoleacetic acid	190.059	99.546	1.621	0.028	0.316
5-Hydroxyindoleacetate	192.065	35.225	1.715	0.029	0.590
Jasmonic acid	193.120	184.510	1.611	0.037	0.614
N-Phenylacetamide	196.095	80.847	1.568	0.042	0.572
Gly-Gln	203.089	245.191	1.281	0.046	0.613
L-Tryptophan	205.095	179.176	1.713	0.018	0.434
4-Aminobutyric acid	207.136	49.432	1.663	0.019	0.692
Ibuprofen	207.136	36.423	1.748	0.003	0.594
Pyridoxal (Vitamin B6)	209.091	85.287	1.445	0.045	0.647
D-Xylose	211.078	210.453	1.738	0.042	0.586
Triethanolamine	213.122	401.241	1.567	0.038	0.675
Desipramine	231.170	51.509	1.905	0.022	0.402
N-Acetylglutamine	233.051	26.581	1.820	0.040	0.586
L-Valine	235.167	49.399	1.779	0.008	0.554
(+)-Muscarine cation	241.106	29.296	1.709	0.029	0.665
D-Mannose	244.076	277.916	1.778	0.014	2.096
Arg-Ser	244.138	438.351	1.650	0.028	0.759
Lamivudine	247.090	165.967	1.669	0.021	0.678
Ile-Thr	255.133	485.522	1.968	0.024	0.341

3-Hydroxyflavone	256.103	233.994	1.467	0.040	0.626
Arg-Cys	260.112	165.375	1.259	0.022	0.533
Phe-Pro	263.138	221.295	1.660	0.016	0.607
Gly-Glu	265.106	228.713	2.113	0.008	0.685
5'-Deoxyadenosine	269.135	234.703	1.327	0.050	0.867
Palmitic acid	274.272	63.210	1.715	0.047	0.383
Tyr-Pro	278.126	27.976	1.626	0.039	0.652
N-.alpha.-Acetyl-L-arginine	280.141	27.976	1.785	0.010	0.517
Gemcitabine	286.056	176.096	1.589	0.015	0.620
N4-Acetylcytidine	286.103	158.691	1.575	0.028	0.545
Nortriptyline	286.163	181.948	1.899	0.006	0.394
Tyr-Gln	292.127	33.609	1.834	0.002	0.425
Trp-Cys	308.108	213.680	1.913	0.006	0.500
Ser-Lys	310.052	338.549	1.544	0.017	0.493
15-Deoxy-delta-12,14-PGJ2	317.208	49.084	1.529	0.042	0.688
2-Oxoadipic acid	321.086	46.427	1.977	0.000	0.360
Ile-Ala-Arg	323.219	49.283	1.757	0.011	0.626
Deoxycoformycin	332.131	272.230	1.532	0.018	0.648
(+-)5,6-DHET	339.250	49.847	1.706	0.022	0.662
Arg-Trp	360.190	230.251	1.363	0.040	0.619
Eicosapentaenoic acid	363.249	49.309	1.865	0.019	0.579
3.alpha.-Mannobiose	365.102	430.633	1.869	0.042	1.319
Fluvoxamine	382.169	149.788	1.685	0.042	0.475
Midazolam	389.092	43.451	1.586	0.027	0.617
Homoveratric acid	415.138	49.096	1.609	0.009	0.668
Cytidine 5'-diphosphocholine (CDP-choline)	489.112	199.244	1.489	0.045	0.708
Cytidine 5'-diphosphocholine (CDP-choline)	496.335	181.366	1.414	0.035	0.596

Table S7. Pathway analysis of urine metabolomics in PRB and CON groups of fattening sheep

Pathway	Total	Hits	Raw P	Impact	Hits compunds
Tryptophan metabolism	41	4	0.009	0.201	L-Tryptophan cpd:C00078; 5-Hydroxyindoleacetic acid cpd:C05635; Oxoadipic acid cpd:C00322; Formylanthranilic acid cpd:C05653
Glycerophospholipid metabolism	29	2	0.121	0.092	Citicoline cpd:C00307; Choline cpd:C00114
Taurine and hypotaurine metabolism	7	1	0.137	0.750	Taurine cpd:C00245
Vitamin B6 metabolism	9	1	0.173	0.490	Pyridoxal cpd:C00250
Valine, leucine and isoleucine biosynthesis	11	1	0.208	0.333	L-Valine cpd:C00183
Biosynthesis of unsaturated fatty acids	42	2	0.217	0	Palmitic acid cpd:C00249; Eicosapentaenoic acid cpd:C06428
Pantothenate and CoA biosynthesis	15	1	0.272	0	L-Valine cpd:C00183
Pentose and glucuronate interconversions	15	1	0.272	0.083	D-Xylose cpd:C00181
Terpenoid backbone biosynthesis	15	1	0.272	0.172	Mevalonic acid-5P cpd:C01107
Propanoate metabolism	20	1	0.346	0	Succinic acid cpd:C00042
Butanoate metabolism	20	1	0.346	0	Succinic acid cpd:C00042
Citrate cycle (TCA cycle)	20	1	0.346	0.026	Succinic acid cpd:C00042
Lysine degradation	20	1	0.346	0.090	Oxoadipic acid cpd:C00322
Alanine, aspartate and glutamate metabolism	23	1	0.386	0	Succinic acid cpd:C00042
Aminoacyl-tRNA biosynthesis	64	2	0.389	0	L-Valine cpd:C00183; L-Tryptophan cpd:C00078
Fatty acid elongation in mitochondria	27	1	0.437	0	Palmitic acid cpd:C00249
Glycine, serine and threonine metabolism	32	1	0.494	0	Choline cpd:C00114
Fatty acid biosynthesis	38	1	0.556	0	Palmitic acid cpd:C00249
Valine, leucine and isoleucine degradation	38	1	0.556	0	L-Valine cpd:C00183
Fatty acid metabolism	39	1	0.565	0	Palmitic acid cpd:C00249

Primary bile acid biosynthesis	46	1	0.627	0.030	Taurine cpd:C00245
Cyanoamino acid metabolism	6	1	0.087	0	L-Serine cpd:C00065
Thiamine metabolism	7	1	0.101	0	Thiamine monophosphate cpd:C01081
Ascorbate and aldarate metabolism	9	1	0.128	0	L-Gulonolactone cpd:C01040
Methane metabolism	9	1	0.128	0.400	L-Serine cpd:C00065
Tyrosine metabolism	42	2	0.130	0	Gentisate aldehyde cpd:C05585; Gentisic acid cpd:C00628
Riboflavin metabolism	11	1	0.155	0.333	Flavin Mononucleotide cpd:C00061
Histidine metabolism	14	1	0.193	0	Carnosine cpd:C00386
Sphingolipid metabolism	21	1	0.275	0	L-Serine cpd:C00065
Galactose metabolism	26	1	0.329	0.005	Uridine diphosphategalactose cpd:C00052
Cysteine and methionine metabolism	28	1	0.350	0.023	L-Serine cpd:C00065
Amino sugar and nucleotide sugar metabolism	37	1	0.435	0	Uridine diphosphategalactose cpd:C00052
Pyrimidine metabolism	37	1	0.435	0.021	Uridine cpd:C00299

Table S8. Analysis of significant differential expression of circRNA between PRB group and CON group

circRNA id[1]	MeanRPKM[2] (PRB)	MeanRPKM (CON)	log2FoldChange[3]	Pvalue[4]
**Up**				
circRNA04879	713.968	0.000	22.767	0.040
circRNA04891	666.931	0.000	22.669	0.039
circRNA00099	593.008	100.101	2.567	0.024
**Down**				
circRNA01712	0.000	175.609	-20.744	0.017
circRNA01506	0.000	470.300	-22.165	0.044
circRNA02476	97.405	196.253	-1.011	0.041
circRNA04326	0.000	355.816	-21.763	0.005

[1] circRNA ID: Transcript number.

[2] MeanTPM: Expression level of grouping.

[3] log2FoldChange: log2 value of difference multiple.

[4] Pvalue: Statistical significance test indicators.

Table S9. Significant differential expression analysis of transcripts (lncRNA, mRNA) between PRB group and CON group

Transcript id[1]	MeanTPM (PRB)	MeanTPM (CON)	log2FoldChange	Pvalue
**Up**				
MSTRG.32443.1	911.183	151.893	2.585	0.000
MSTRG.34857.9	6.250	0.000	15.932	0.000
MSTRG.96967.4	9.373	0.243	5.268	0.000
MSTRG.34857.10	9.733	0.000	16.571	0.000
MSTRG.61223.7	11.583	2.093	2.468	0.000
MSTRG.86362.1	41.113	0.000	18.649	0.000
ENSOART00000019541	7.213	1.567	2.203	0.000
ENSOART00000008854	23.977	1.057	4.504	0.000
ENSOART00000002985	5.913	0.833	2.827	0.000
**Down**				
MSTRG.16260.13	0.000	5.653	-15.787	0.000
MSTRG.16260.15	0.000	10.920	-16.737	0.000
ENSOART00000016500	261.843	706.157	-1.431	0.000
ENSOART00000000735	0.000	7.543	-16.203	0.000
ENSOART00000004171	0.000	6.160	-15.911	0.000
ENSOART00000014309	0.107	10.300	-6.593	0.000
ENSOART00000008853	0.003	21.480	-12.654	0.000
ENSOART00000007695	15.077	33.660	-1.159	0.000
ENSOART00000014234	0.050	37.867	-9.565	0.000
ENSOART00000020258	48.740	141.280	-1.535	0.000
ENSOART00000013884	0.180	73.297	-8.670	0.000

[1]Transcript ID：Transcript number.
